# Prevalence and Risk Factors of Allergic Rhinitis Among the Population in the Makkah Region, Saudi Arabia: A Cross-Sectional Study

**DOI:** 10.7759/cureus.34863

**Published:** 2023-02-11

**Authors:** Sanad M Alharthi, Faisal M Alzahrani, Saad M Alharthi, Abdulrahman F Kabli, Abdulrahman A Baabdullah, Emad A Baatiyyah, Alwaleed S Alzahrani, Badr S Almuqati, Mokhtar M Shatla

**Affiliations:** 1 Medicine and Surgery, Umm Al-Qura University, Makkah, SAU; 2 Family Medicine, Umm Al-Qura University, Makkah, SAU

**Keywords:** allergic rhinitis, practices towards ar, knowledge of allergic rhinitis, risk factors of allergic rhinitis, ar, prevalence of allergic rhinitis

## Abstract

Background

Allergic rhinitis (AR) is considered a high global disease burden; hence, the shortage of knowledge would lead to poor adherence to management and preventive measures and increase the exacerbation of AR symptoms. This study aimed to evaluate the prevalence and risk factors, and assess the knowledge and practices of the population regarding AR among the population in Makkah city, Saudi Arabia.

Methodology

This was an online survey cross-sectional study conducted in December 2022 via social media platforms (WhatsApp and Twitter) to collect data on participants’ demographics, prevalence, risk factors, knowledge, and attitudes toward AR, using a validated Arabic version of a self-administered questionnaire.

Results

The study involved 466 participants. Of the participants, 55.8% were aged 31 to 45 years, and 286 (61.4%) were females. The prevalence of AR among the participants was 45%. The most common symptoms were a blocked nose (79.6%), sneezing (74.2%), and a runny nose (71.5%). Furthermore, the prevalence of rhinoconjunctivitis in this study was 56%. The most common inhalant allergen was house dust (73.0%). Only the age and history of asthma or eczema were significant factors associated with AR. Overall, most of the subjects (94.85%) had adequate knowledge regarding AR.

Conclusion

A high prevalence of AR was observed. Understanding the factors linked with AR is imperative to ensure better adherence to preventive management plans.

## Introduction

Allergic rhinitis (AR) is a non-communicable disorder of nasal hypersensitivity that occurs in response to an allergen that activates the immune system causing inflammation. The cardinal nasal symptoms of AR are rhinorrhea, sneezing, nasal congestion, and itching. They diminish spontaneously or after medical treatment [1,2]. The major risk factors related to AR are genetic, environmental, social, and home pets [3].

A study reported that the prevalence of AR was 15-25% across Asia, Europe, the Americas, and Africa [2]. Its prevalence is significantly increasing in Saudi Arabia [4].

One study involving 900 Saudi adults reported an AR prevalence of 34% [5]. In the Madinah population of Saudi Arabia, this prevalence was reported to be 27.9% amongst 524 sampled participants [6].

AR is a global health burden. Despite being a non-severe illness, it is affecting social life, work, and academic performance [7,8]. The effective treatment of AR is the identification and prevention of exposure to the allergen. A disparity between the knowledge and practices regarding AR was noted in Saudi Arabia, resulting in a poor quality of life [9].

The epidemiologic data on AR in the Makkah region of Saudi Arabia is inadequate. Therefore, this study aimed to determine the prevalence of AR among the population of the Makkah region, Saudi Arabia. Moreover, we assessed the knowledge and attitudes regarding AR and identified common allergen triggers and risk factors in this population.

## Materials and methods

A cross-sectional study was conducted in December 2022 by posting an online survey on social media platforms to collect data on participants’ demographics and the prevalence, knowledge, and attitudes toward AR among the residents in the Makkah region, Saudi Arabia, using a validated Arabic version of a self-administered questionnaire [6].

The sample size of this study was determined according to the Raosoft calculator (Raosoft, Inc., Seattle, WA) to be more than 385 participants, considering a confidence interval of 95% and the level of significance (p-value) at 5%. Randomly, 466 participants replied to the questionnaire. Individuals who were above 16 years and lived in the Makkah region were included.

The questionnaire comprised items to collect participants’ demographic data, previous history of AR, results of allergic test if previously done, the presence of allergenic symptoms in the last 12 months, its triggering factors and the season of its occurrence, the type of medications used for relief of the symptoms of AR, and lastly, the knowledge and attitude toward AR.

SPSS statistics version 22 (IBM Corp., Armonk, NY) was used to analyze the data. Statistical significance was described as a p-value < 0.05. Categorical variables are represented as frequencies (percentages). The chi-square test was conducted to examine the relationships between different demographic data and the prevalence of AR for bivariate analyses. Concerning knowledge questions (n = 7), a score of “1” was given to correct answers while a score of “0” was given to incorrect answers. The overall score and its percentage were calculated. Individuals who scored less than 50% were considered to have “inadequate knowledge” and those who scored 50% and more were considered to have “adequate knowledge.”

The Biomedical Research Ethics Committee of Umm Al-Qura University authorized this study (IRB number: HAPO-02-K-012-2022-11-1329).

## Results

This survey was performed among the population of the Makkah region of Saudi Arabia. We explored the presence of AR in 466 participants. Table 1 summarizes the participants’ demographic characteristics. The age representation of the respondents was as follows: 16-30 years (34.5%), 31-45 years (55.8%), and above 46 years (9.7%). Females consisted of 61.4%. Almost 90% of the subjects were Saudi nationals and 55.4% had a university/high education. Nearly 59.7% of the subjects were unemployed. The vast majority (83.7%) were nonsmokers.

**Table 1:** Demographic characteristics of the participants (n = 466)

Variables		Frequency	Percent
Age	From 16 to 30 years	161	34.5
	From 31 to 45 years	260	55.8
	More than 46 years	45	9.7
Gender	Male	180	38.6
	Female	286	61.4
Nationality	Saudi	419	89.9
	Non-Saudi	47	10.1
Educational level	Primary school	14	3.0
	Intermediate school	26	5.6
	Secondary school	168	36.1
	University/high education	258	55.4
Employment	Employed	188	40.3
	Non-employed	278	59.7
Smoking	Yes	76	16.3
	No	390	83.7

The prevalence of AR among the participants was 45.0%. More than two-thirds of the subjects had sneezing (74.2%), a runny nose (71.5%), and a blocked nose (79.6%) within the past year, excluding a common cold. Itchy, watery eyes occurred in 56.0% of the participants. These symptoms were more frequent in winter (30.0%), followed by summer (29.4%). House dust was the most mentioned trigger factor for these symptoms (73%). Around two-thirds (73.0%) of the participants had a lack of sleep due to the debilitating symptoms. More than two-thirds of the subjects (70.2%) believed AR to be allergic. Nevertheless, just 29.4% of the participants had been investigated for allergies; among them, half were positive for the allergy test. About one-third of the participants (33.3%) stated to have been diagnosed with asthma or eczema. A family history of allergy was declared by the majority of the participants (68.2%) (Table 2).

**Table 2:** Responses to questions related to the history of allergic rhinitis

Variables		Frequency	Percent
In the past 12 months, have you had sneezing apart from the common cold?	Yes	346	74.2
	No	120	25.8
In the past 12 months, have you had a runny nose apart from the common cold?	Yes	333	71.5
	No	133	28.5
In the past 12 months, have you had a blocked nose apart from the common cold?	Yes	371	79.6
	No	95	20.4
If yes, at least one nose problem in the past 12 months, was this nose problem accompanied by itchy watery eyes?	Yes	261	56.0
	No	205	44.0
In which season did this nose problem occur?	Winter	140	30.0
	Spring	55	11.8
	Summer	137	29.4
	Autumn	85	18.2
	Not happened	49	10.5
What trigger factors provoke or increase your problem?	House dust	340	73.0
	House dust mites	10	2.1
	Pollens	3	0.6
	Perfumes and bukhoor	43	9.2
	Animals (cat, dog, etc.)	11	2.4
	Others	59	12.6
Does allergic rhinitis affect the quality of life in terms?	Lack of sleep	340	73.0
	Poor concentration	72	15.5
	Absence from work	18	3.9
	Social isolation	36	7.7
Do you think to be allergic?	Yes	327	70.2
	No	139	29.8
Have you already been tested for allergy (skin prick tests, immunoglobulin E, etc.)?	Yes	137	29.4
	No	329	70.6
If yes, what was the result?	Positive	59	12.7
	Negative	57	12.2
	Not done	350	75.1
Has a doctor already diagnosed that you suffer/suffered from asthma or eczema?	Yes	155	33.3
	No	311	66.7
Is any member of your family suffering from asthma, eczema, or allergic rhinitis?	Yes	318	68.2
	No	148	31.8
If yes, who and what disease? Mother	Asthma	100	21.5
	Allergic rhinitis	61	13.1
	Eczema	38	8.2
If yes, who and what disease? Father	Asthma	53	11.4
	Allergic rhinitis	54	11.6
	Eczema	28	6.0
If yes, who and what disease? Siblings	Asthma	129	27.7
	Allergic rhinitis	70	15.0
	Eczema	31	6.7

The bivariate analysis showed that there were only significant associations between AR and age (p = 0.033) and the presence of asthma or eczema (p = 0.000) (Table 3).

**Table 3:** The bivariate analysis of allergic rhinitis

Variables		Have you been diagnosed by a doctor with allergic rhinitis?		
		Yes, n (%)	No, n (%)	P-value
Gender	Male	74 (41.1)	106 (58.9)	0.174
	Female	136 (47.6)	150 (52.4)	
Age	From 16 to 30 years	61 (37.9)	100 (62.1)	0.033
	From 31 to 45 years	123 (47.3)	137 (52.7)	
	More than 46 years	26 (57.8)	19 (42.2)	
Nationality	Saudi	195 (46.5)	224 (53.5)	0.056
	Non-Saudi	15 (31.9)	32 (68.1)	
Educational level	Primary school	4 (28.6)	10 (71.4)	0.417
	Intermediate school	13 (50)	13 (50)	
	Secondary school	71 (42.3)	97 (57.7)	
	University/high education	122 (47.3)	136 (52.7)	
Job	Employed	89 (47.3)	99 (52.7)	0.417
	Non-employed	121 (43.5)	157 (56.5)	
Are you a smoker?	Yes	27 (35.5)	49 (64.5)	0.068
	No	183 (46.9)	207 (53.1)	
Has a doctor already diagnosed that you suffer/suffered from asthma or eczema?	Yes	97 (62.6)	58 (37.4)	0.000
	No	113 (36.3)	198 (63.7)	
Is any member of your family suffering from asthma, eczema, or allergic rhinitis?	Yes	151 (47.5)	167 (52.5)	0.124
	No	59 (39.9)	89 (60.1)	

Regarding the knowledge of AR, 95.9% of the respondents understood that the classic symptoms of AR are nasal congestion, nasal itch, rhinorrhea, and sneezing. Moreover, they knew that the disease could be prevented by avoiding triggering factors (92.1%). Most (84.8%) of the subjects had a good knowledge of the definition of AR. The majority (83.5%) realized that nasal sprays could be used for reducing AR symptoms. The majority (82%) agreed that AR was not a contagious disease. On the other hand, half of the participants (51.9%) knew that AR is a genetically inherited disease and is linked to asthma and conjunctivitis (49.6%) (Table 4). Overall, most of the participants (94.85%) had adequate knowledge of AR.

**Table 4:** Right responses to knowledge statements about allergic rhinitis AR: allergic rhinitis.

	Frequency	Percent
Allergic rhinitis is a contagious disease (no)	342	82.0
Allergic rhinitis is a hypersensitivity of the nasal mucosa (yes)	395	84.8
AR could be prevented by avoiding triggering factors (yes)	429	92.1
Nasal sprays could be used for reducing the symptoms of AR (yes)	389	83.5
It is a genetically inherited disease (yes)	242	51.9
AR is linked to asthma and conjunctivitis (yes)	231	49.6
The classic symptoms of AR are nasal congestion, nasal itch, rhinorrhea, and sneezing (yes)	447	95.9

Regarding the practices of the participants toward the disease, 42.7% of the participants visited a physician when developing symptoms, while 38.4% used steroid nasal sprays. Nearly half of the respondents purchased over-the-counter drugs without consulting a physician. Most of the respondents (83.7%) avoided house dust and smoke and 71.2% strictly followed the doctor's instructions (Table 5).

**Table 5:** Practice patterns of the participants regarding allergic rhinitis

		Frequency	Percent
Usually, visit a physician when developing symptoms	Yes	199	42.7
	No	267	57.3
I may use steroid nasal sprays	Yes	179	38.4
	No	287	61.6
I buy over-the-counter drugs without consulting a physician	Yes	250	53.6
	No	216	46.4
I avoid house dust and smoke	Yes	390	83.7
	No	76	16.3
I strictly follow the doctor’s instructions	Yes	332	71.2
	No	134	28.8

## Discussion

AR is a major burden on patients that affects their quality of life in all aspects [10]. This study assessed the prevalence of AR, evaluated individuals' knowledge and practice about the disease, and explored the most frequent triggering factors of AR among the residents of the Makkah region, Kingdom of Saudi Arabia.

The prevalence of AR was reported to be 45.0% among the participants in this survey study. This finding was higher than that of previously published studies. A recent study done in Al Baha city, Saudi Arabia reported a slightly lower prevalence: 32% and 38.6% in males and females, respectively [11]. A study done in Riyadh, Saudi Arabia, which involved children, found a prevalence of AR of 26.5% [9]. Conversely, a study conducted among students of the University of Hail in Saudi Arabia found a prevalence of AR of 51% [12]. Moreover, the prevalence varied between 15% and 25% in a study including different geographical areas (Europe, Asia, the Americas, and Africa) [13]. From the results of the present study and those of other studies mentioned, AR is a frequent issue with variable impacts in different countries, related to environmental factors.

Our data revealed that most nasal symptoms were more noted in winter in comparison to other seasons, corroborating the findings of previous studies [11,14]. As described in the present study, house dust is the most frequently reported triggering factor for AR. Other studies verify this finding, illuminating that the indoor living style in Saudi Arabia has been ascribed to AR [11,15].

Contrary to the previous studies, most of the individuals in the present study had adequate knowledge of AR [5,16]. In this perspective, comparison between different studies is impractical because of the various instruments used to assess knowledge.

In our result, the practice of the participants toward AR was similar to those reported by the previous studies conducted in Saudi Arabia and Canada [11,17].

This study had several limitations. First, the cross-sectional design confirms only association. Second, the limit to a specific city could influence the generalizability of the results. Third, we explored the prevalence of AR in adults, and this may not represent the actual prevalence in all age groups. Nevertheless, this study presents public health significance in exploring the magnitude, determinants, knowledge, and practice associated with a common disease influencing the quality of life in our society.

## Conclusions

This study revealed a high prevalence of AR among the residents of Makkah, Saudi Arabia. More consideration should be given to AR in this area. House dust was the most important allergen cause among participants. Appropriate recognition and evaluation of the disease is important and could guarantee early management and improvement of the quality of life.
